# Loss of PTEN induces lung fibrosis via alveolar epithelial cell senescence depending on NF‐κB activation

**DOI:** 10.1111/acel.12858

**Published:** 2018-12-12

**Authors:** Yaqiong Tian, Hui Li, Ting Qiu, Jinghong Dai, Yingwei Zhang, Jingyu Chen, Hourong Cai

**Affiliations:** ^1^ Department of Respiratory Medicine The Affiliated Drum Tower Hospital of Nanjing University Medical School Nanjing China; ^2^ Department of Respiratory Medicine KunShan Hospital of Traditional Chinese Medicine Kunshan China; ^3^ Jiangsu Key Laboratory of Organ Transplantation, Wuxi People’s Hospital Nanjing Medical University Wuxi China

**Keywords:** aging, cellular senescence, idiopathic pulmonary fibrosis, nuclear transcription factor‐κB, phosphatase and tension homolog deleted on chromosome ten, senescence‐associated secretory phenotype

## Abstract

Idiopathic pulmonary fibrosis (IPF) is an aging‐associated disease with poor prognosis. Currently, there are no effective drugs for preventing the disease process. The mechanisms underlying the role of alveolar epithelial cell (AEC) senescence in the pathogenesis of IPF remain poorly understood. We aimed to explore whether PTEN/NF‐κB activated AEC senescence thus resulting in lung fibrosis. First, we investigated the association between the activation of PTEN/NF‐κB and cellular senescence in lung tissues from IPF patients. As a result, decreased PTEN, activated NF‐κB and increased senescent markers (P21^WAF1^, P16^ink4a^, and SA‐β‐gal) were found in AECs in fibrotic lung tissues detected by immunohistochemistry (IHC) and immunofluorescence (IF). In vitro experiments showed increased expression levels of senescent markers and augmented senescence‐associated secretory phenotype (SASP) in AECs treated with bleomycin (Blm); however, PTEN was reduced significantly following IκB, IKK, and NF‐κB activation after stimulation with Blm in AECs. AEC senescence was accelerated by PTEN knockdown, whereas senescence was reversed via NF‐κB knockdown and the pharmacological inhibition (BMS‐345541) of the NF‐κB pathway. Interestingly, we observed increased collagen deposition in fibroblasts cultured with the supernatants collected from senescent AECs. Conversely, the deposition of collagen in fibroblasts was reduced with exposure to the supernatants collected from NF‐κB knockdown AECs. These findings indicated that senescent AECs controlled by the PTEN/NF‐κB pathway facilitated collagen accumulation in fibroblasts, resulting in lung fibrosis. In conclusion, our study supports the notion that as an initial step in IPF, the senescence process in AECs may be a potential therapeutic target, and the PTEN/NF‐κB pathway may be a promising candidate for intervention.

## 1 INTRODUCTION

Idiopathic pulmonary fibrosis (IPF) is a chronic, progressive, irreversible, and fatal disease with unknown cause. There are limited choices for treatment, and the prognosis is poor. Epidemiological investigations and clinical observations indicate that IPF is an aging‐related disease (Ley & Collard, 2013; Sueblinvong et al., 2012) because it occurs frequently in middle‐aged and elderly adults. Most patients are over 60 years old at initial diagnosis**,** and IPF morbidity and mortality increase with aging.

At present, reasonable opinion suggests that alveolar epithelial cell (AEC) injury and abnormality in repair procedures play key roles in the genesis and development of IPF. AEC injury can break down the integrity of the epithelial barrier. As a feedback mechanism, AECs are activated aberrantly. However, sustained AEC injury causes epithelial apoptosis. Another potential response to injury in AECs is the occurrence of premature senescence (Chilosi, Carloni, Rossi, & Poletti, 2013). The accelerated senescence of AECs is one of the mechanisms that drives the aberrant activation of AECs (Yanagi, Tsubouchi, Miura, Matsumoto, & Nakazato, 2015). Abnormally activated cells induce fibroblasts and myofibroblasts to secrete redundant amounts of extracellular matrix, resulting in the deposition of collagen and destruction of the lung architecture (King, Pardo, & Selman, 2011). The levels of senescence‐associated markers, such as P16^INK4a ^and SA‐β‐Gal, are higher in the AECs of IPF lung tissues than in normal lung tissues (Minagawa et al., 2011; Schafer et al., 2017).

The pathogenesis of IPF and cancer shares some similarities (Buendía‐Roldán, Mejía, Navarro, & Selman, 2017). Phosphatase and tension homolog deleted on chromosome ten (PTEN), a tumor suppressor gene, is also involved in the onset and development of IPF. PTEN is a multifunctional molecule expressed in various cells. It performs its function by negatively regulating several signal transduction pathways, primarily the PI3K/Akt pathway, to regulate cell growth, proliferation, apoptosis, and adherence (Xia et al., 2010). Importantly, NF‐κB, an important target of the PI3K/Akt pathway, regulates the secretion of many cytokines (Cheng, Lee, Lin, Hsiao, & Yang, 2014). PTEN signaling plays critical roles in normal mouse lung morphogenesis, bronchoalveolar stem cell homeostasis, and the prevention of lung adenocarcinoma (Yanagi et al., 2007) and regulates fibroblasts in lung fibrosis (White et al., 2006). The loss of PTEN leads to the destruction of AEC integrity and alveolar basement membrane, thus contributing to acute lung injury and fibrosis in a mouse lung fibrosis model (Miyoshi et al., 2013). Therefore, we presumed that the abnormal expression of PTEN might be involved in the genesis and development of IPF; however, the mechanisms remain to be elucidated.

Cellular senescence is a condition of irreversible cell growth arrest, meaning that senescent cells remain alive while possessing the senescence‐associated secretory phenotype (SASP). In other words, senescent cells have the ability to express diverse cytokines, growth factors, and proteases to maintain cell growth arrest and promote the degeneration and hyperplasia of neighboring cells in a paracrine manner (Coppe, Desprez, Krtolica, & Campisi, 2010; Kuilman & Peeper, 2009). As a consequence, cellular senescence participates in the onset and development of various kinds of aging‐related diseases. Recently, researchers found that senolytic drugs that are selectively induce senescent cells accumulated in tissues to apoptosis have the ability to partly prevent or reverse the process of aging‐related chronic diseases including pulmonary fibrosis (Kirkland, Tchkonia, Zhu, Niedernhofer, & Robbins, 2017). However, the pathogenesis of senescence involved in IPF is largely unknown. PTEN is an important regulator executing its role on cell proliferation and cell cycle; thus, it might be involved in the pathogenesis of senescence‐associated molecular mechanism of IPF.

The NF‐κB signaling pathway is the primary mediator of the appearance of SASP (Coppe et al., 2010; Kuilman & Peeper, 2009; Salminen, Kauppinen, & Kaarniranta, 2012). However, whether PTEN regulates the NF‐κB pathway during AEC senescence and how SASP is involved in the pathogenesis of pulmonary fibrosis remains poorly understood.

In the present study, we confirmed that AECs possess senescent properties in IPF patients**, **in a Blm‐induced mouse pulmonary fibrosis model, and in Blm‐stimulated AECs in vitro. Based on these models, we found that the loss of PTEN led to the activation of NF‐κB, thus participating in the pathogenesis of IPF. Our evidence suggested that supernatants from senescent AECs promoted redundant collagen deposition in fibroblasts and that the knockdown of NF‐κB in AECs reversed this change.

## 2 RESULTS

### 2.1 IPF is an aging‐related disease

**Figure 1:**
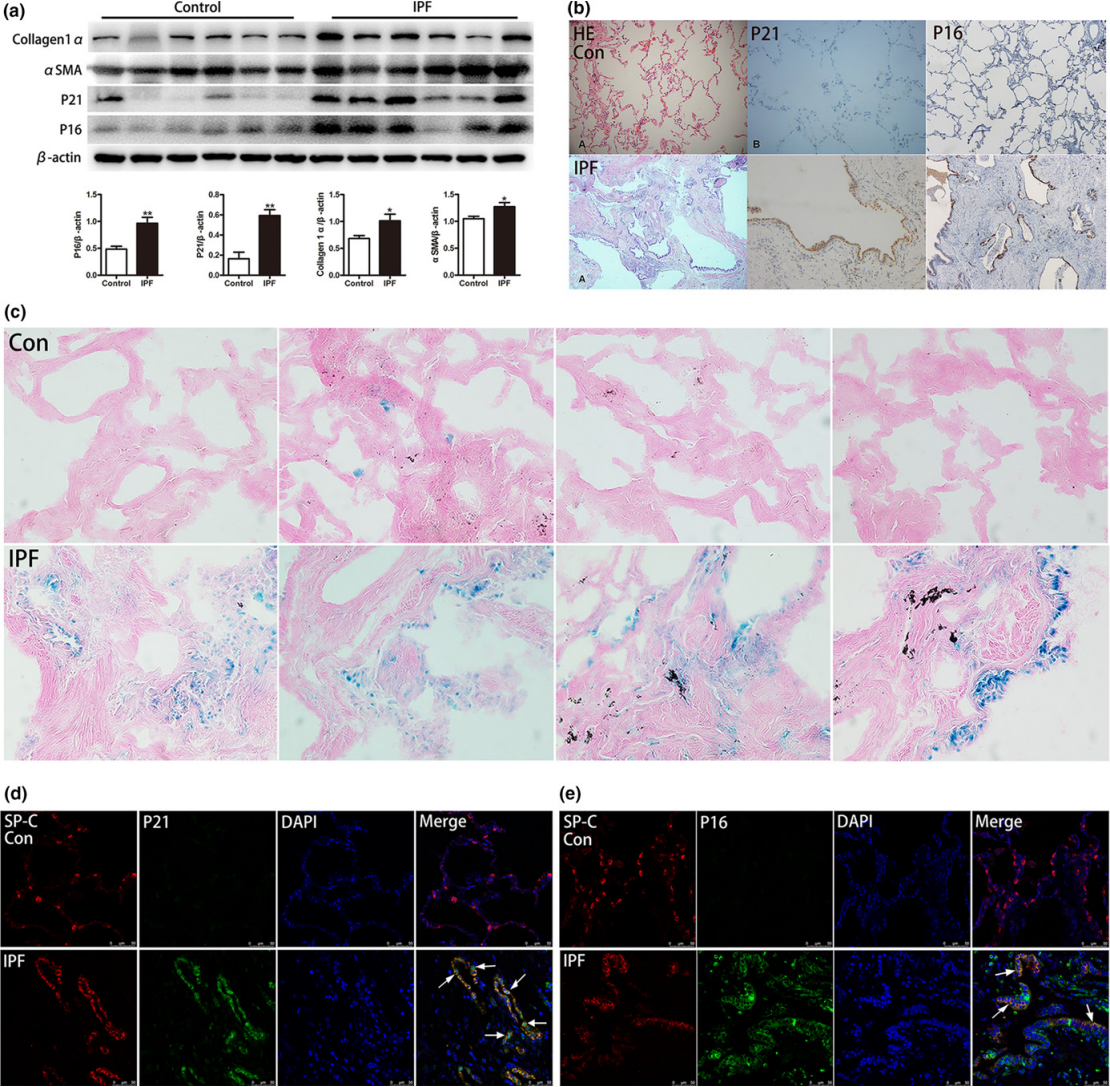
Senescent markers are significantly overexpressed in the lung tissues of IPF patients**.** (a) Senescent markers (P16^ink4a^ and P21^WAF1^) and fibrotic markers (α‐SMA and collagen1α) in IPF lung tissues were measured by western blot. (b) Representative pictures of HE and P21^WAF1^, P16^ink4a^ IHC staining of normal lung tissue, and lung tissues from IPF patients (original magnification, 200×). (c) Representative results of SA‐β‐Gal staining of human lung tissues (original magnification, 400×). (d, e) Immunofluorescence staining of SP‐C (an AEC2‐specific marker, red) and P21^WAF1^, P16^ink4a^ (green) were conducted to confirm senescent markers mainly expressed in AECs (original magnification, 400×). Data are shown as the mean ± *SEM*. *p < 0.05, **p < 0.005. Unpaired, two‐tailed Student's *t* test

In our study, Senescent markers P21^WAF1^, P16^ink4a^, and SA‐β‐Gal were used to detect the appearance of senescence in IPF lung tissues and in AECs. The expression of P16^ink4a^ and P21^WAF1^ was detected by western blot. As shown in Figure 1, we found that the levels of both senescent markers, P16^ink4a^ and P21^WAF1^, and fibrotic markers, collagen1α and αSMA, were greater in the lung tissues from IPF patients than normal lung tissues (Figure 1a). Using HE and IHC staining methods, we found that the alveolar structure was destroyed and that the positive staining of P16^ink4a^ and P21^WAF1 ^was primarily located in AECs in the lung tissues of IPF patients (Figure 1b). In addition, strongly positive SA‐β‐Gal staining primarily located in AECs was observed in IPF lung tissues, whereas it was hardly seen in normal lung tissues (Figure 1c). Furthermore, immunofluorescence was used to examine the spatial location of senescent markers and AEC2 marker (SP‐C), in lung tissues from IPF patients and normal tissues. We observed that both P21^WAF1^ and P16^ink4a^ were overexpressed in IPF lung tissue and were barely found in normal lung tissue. The two senescent markers primarily expressed within AEC2, and P21^WAF1^ located in both nuclear and cytoplasm, whereas P16^ink4a^ mainly expressed in cytoplasm (Figure 1d,e). Taken together, these data suggest that IPF is an aging‐related disease as previous findings demonstrated, and abnormal senescence occurs in AECs in IPF lung tissues.

### 2.2 PTEN/NF‐κB pathway is activated in IPF

**Figure 2:**
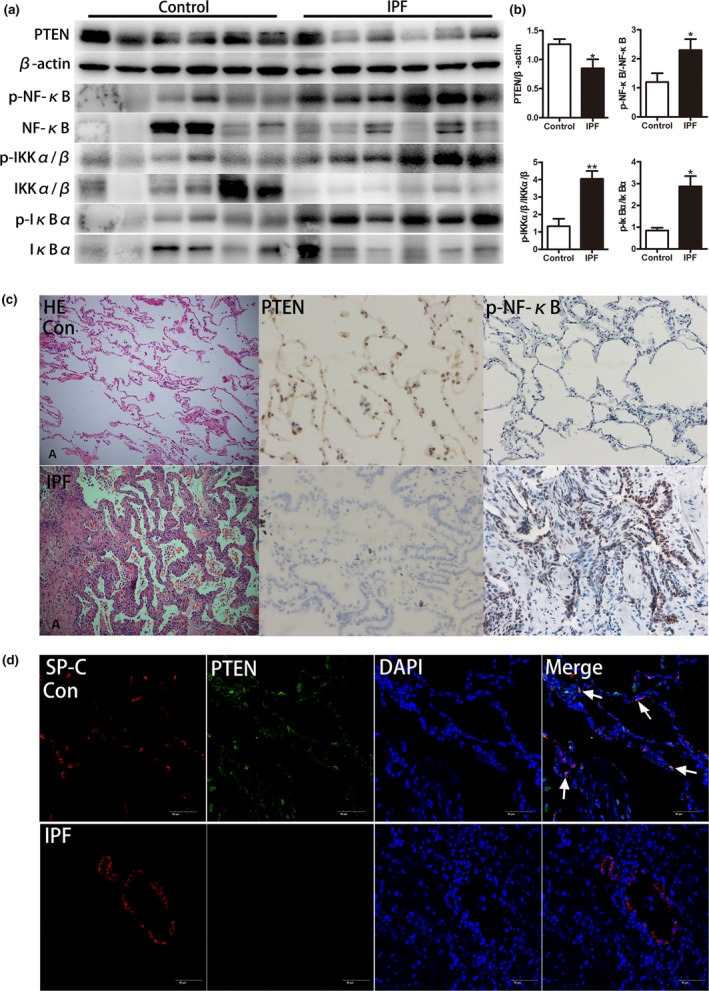
Loss of PTEN and activated NF‐κB pathway in lung tissue from IPF patients**.** (a, b) Western blot was applied to detect the expression of PTEN and activation of IKK, IκB, and NF‐κB. (c) Representative results of HE staining and IHC staining for PTEN, p‐NF‐κB in normal lung and IPF patient lung (original magnification, 200×). (d) Immunofluorescence staining for both SP‐C (red) and PTEN (green) was conducted to examine the spatial distribution of PTEN (original magnification, 200×). Data are shown as the mean ± *SEM*. *p < 0.05, **p < 0.005. Unpaired, two‐tailed Student's *t* test

NF‐κB stays inactive in the cytoplasm because of the inhibitory subunit of NF‐κB, namely, IκBα. The phosphorylation of IκBα leads to its degradation via the proteasome pathway, leading to the activation of NF‐κB in the cytoplasm. Upstream, IκBα is phosphorylated by active IκBα kinase (IKK), a complex consisting of IKKα, IKKβ, and IKKγ. The phosphorylation of IKKβ at serine residues 177 and 181 activates the complex. Unanchored NF‐κB freely translocates to the nucleus and binds to the promoters of its target genes (Solt & May, 2008). We tested the phosphorylation levels of NF‐κB, IKKα/β, IκBα, and PTEN in total protein lysates from the lung tissues of IPF patients. As indicated in Figure 2a,b, the phosphorylation levels of NF‐κB, IKKα/β, and IκBα were higher in IPF lung tissues than in normal tissues, and level of PTEN was lower in IPF. Then, IHC staining was performed in IPF lung tissues and normal lung tissues. The results of PTEN and p‐NF‐κB were according with western blot, and they predominantly distributed within alveolar walls (Figure 2c). To further confirm PTEN location, immunofluorescence staining was conducted. As shown in Figure 2d, we observed that PTEN was down‐regulated in IPF lung tissues. In normal lung tissues, PTEN mainly distributed in AEC and some of PTEN co‐localized with SP‐C (white arrows). Therefore, we suspected that the low expression of PTEN might be related to NF‐κB activation in IPF and that this regulation primarily occurred within AECs. Next, in vitro experiments were performed to further confirm our findings in patients and to elucidate the mechanisms underlying AEC senescence.

### 2.3 Bleomycin induces AEC senescence, and SASP increases collagen expression in fibroblasts

**Figure 3:**
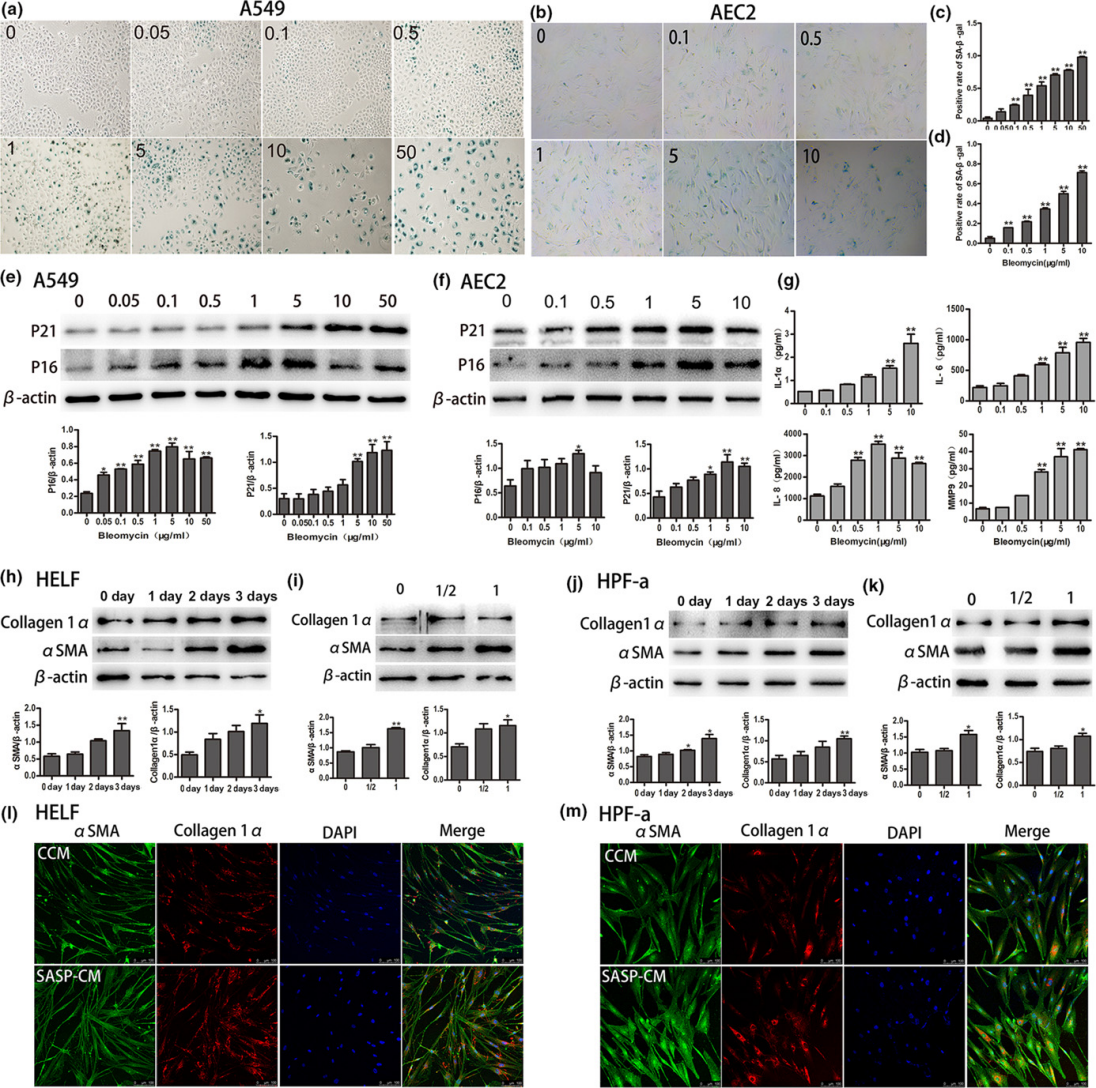
Bleomycin induces AECs senescence, and the supernatants of senescent AECs augment collagen deposition in fibroblasts**.** (a–d) A549 and AEC2 were treated with various concentrations of bleomycin for 5 days. SA‐β‐gal staining was performed to detect cellular senescence (original magnification, 400×). (e, f) The expression of aging‐related markers (P16^ink4a^ and P21^WAF1^) was detected in A549 and AEC2. (g) A549 was stimulated by various concentrations of bleomycin for 5 days, the supernatants were collected, and the levels of SASP cytokines (IL‐1α, IL‐6，and IL‐8) and MMP9 were measured by ELISA. (h–k) After bleomycin (5 µg/ml) was added to induce A549 senescence for 3 days, the medium was replaced by fresh medium without bleomycin to culture another 3 days, then the supernatants were collected to culture HELF in various time points (0,1, 2, and 3 days) and different concentration (1/2‐fold and onefold). Then, the expression of fibrotic markers, collagen1α, and α‐SMA were investigated by western blot. (l, m) Immunofluorescence to fibrotic markers, collagen1α (red), and α‐SMA (green) both in HELF and HPF‐a were conducted after they were cultured in both CCM and SASP‐CM for 3 days (original magnification, 400×). Data are shown as the mean ± *SEM*, *n* ≥ 3 per group. *p < 0.05, **p < 0.005. One‐way ANOVA analysis followed by Dunnett's Multiple Comparison Test

In previous studies, bleomycin was applied to induce AEC senescence (Aoshiba, Tsuji, & Nagai, 2003; Kasper & Barth, 2009). Here, we employed bleomycin to build a cellular senescence model using rat primary AEC2 and A549 cell lines. A549 is usually used as a replacement for primary AECs because of AECs are difficult to obtain and maintain in culture ex vivo. Several cytokines, IL‐1α, IL‐6, IL‐8, and matrix metalloproteinase 9 (MMP9), were tested to evaluate SASP in this study (Coppe et al., 2010; Kuilman & Peeper, 2009). Gradually increasing concentrations of bleomycin were added to the culture medium to stimulate AEC2 and A549 for 5 days, and SA‐β‐Gal staining and western blotting were performed. As shown in Figure 3, we found that the intensity of positive SA‐β‐Gal staining increased along with the increased concentration of bleomycin (Figure 3a–d). In addition, senescence‐related markers P21^WAF1^ and P16^ink4a^ were both increased in a dose‐dependent manner in stimulated AEC2 and A549 (Figure 3e,f). Cytokines and MMP9 expression in bleomycin‐stimulated A549 culture supernatants were detected using ELISA. All measured cytokines and MMP9 levels were greater along with bleomycin accumulation (Figure 3g). To further confirm whether senescent AECs participated in the pathogenesis of IPF through SASP, the supernatants of senescent A549 without bleomycin were collected to culture HELF (human embryonic lung fibroblast) and HPF‐a (Human Pulmonary Fibroblasts‐adult) for 3 days. Then, the total protein lysates of HELF and HPF‐a were obtained to detect the expression of fibrotic markers (collagen 1α and αSMA) by western blot and immunofluorescence. Interestingly, we found that both collagen1α and αSMA were increased along with the culture time of senescent supernatants and concentration of senescent supernatants (Figure 3h–k). After stimulation of senescent supernatants for 3 days, both HELF and HPF‐a, contrast with control conditioned medium (CCM) group, the expression both collagen1α and αSMA increased in senescent conditioned medium (SASP‐CM) group (Figure 3l,m). These results, identical to those of previous studies, indicated that bleomycin was effective in inducing AEC senescence in vitro. Senescent AECs may promote collagen deposition in HELF through SASP.

### 2.4 PTEN is reduced in senescent AECs, whereas NF‐κB is activated

**Figure 4:**
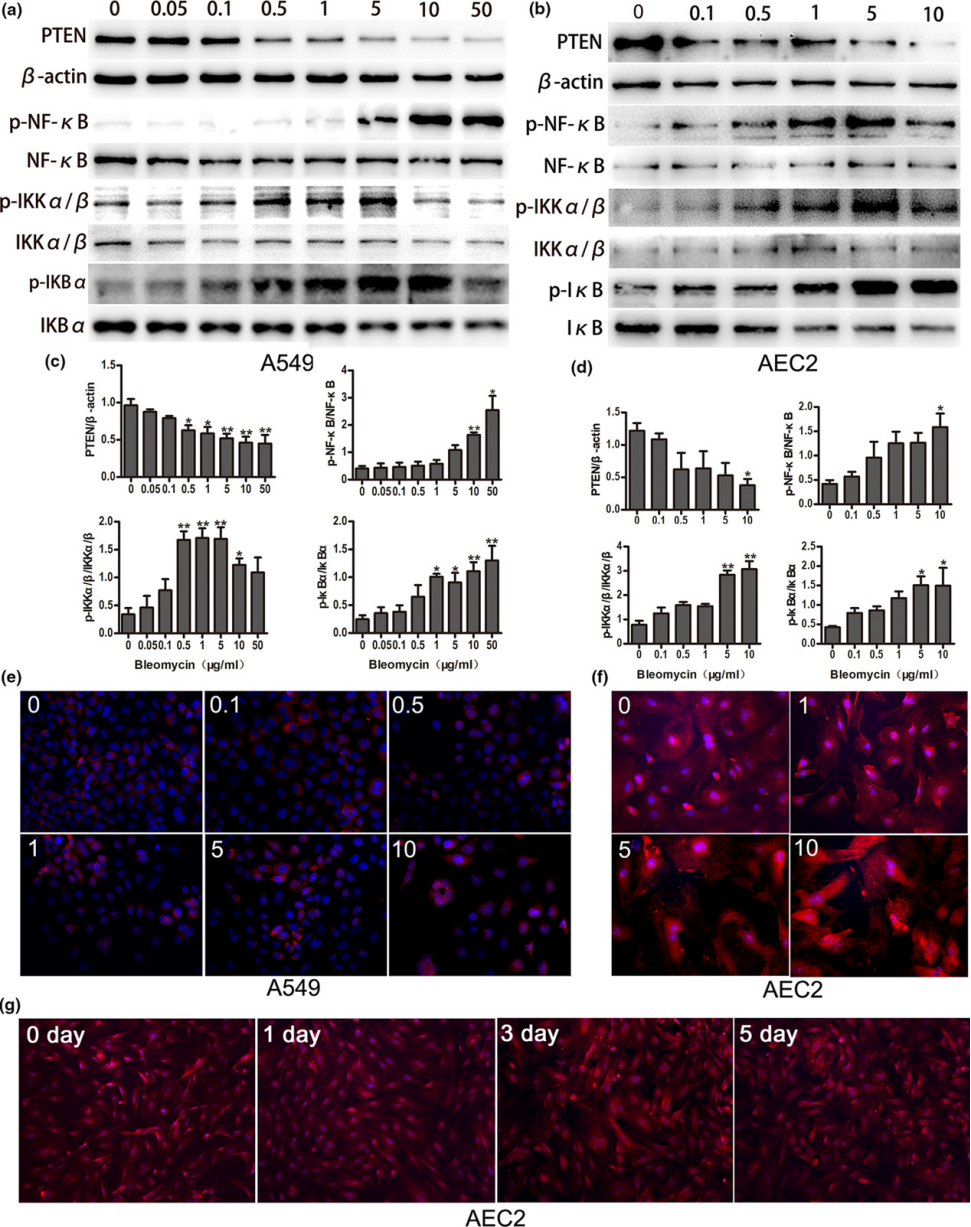
Decreased PTEN and the activation of NF‐κB pathway in senescent A549 and AEC2**. **(a–d) Gradually increased concentrations of bleomycin were added to culture medium of A549 and AEC2 for 5 days, and total protein was extracted. PTEN expression and activation of IKK, IκB and NF‐κB were detected. (e and f) A549 and AEC2 were cultured in various concentrations of bleomycin for 5 days. Immunofluorescence staining for p‐NF‐κB (red) was performed to confirm its location and production (original magnification, 400×). (g) After isolation from rat lung, ACE2 was cultured for various periods, and immunofluorescence staining for SP‐C (AEC2‐specific marker, red) was conducted to identify the purity of AEC2 (original magnification, 200×). Data are shown as the mean ± *SEM*, *n* ≥ 3 per group. *p < 0.05, **p < 0.005. One‐way ANOVA analysis followed by Dunnett's Multiple Comparison Test

To investigate the role of PTEN and the NF‐κB pathway during cellular senescence, we examined the expression of PTEN and NF‐κB in both bleomycin‐induced senescent rat primary AEC2 and A549. As shown in Figure 4, the activation of the NF‐κB pathway, including the phosphorylation of IKKα/β, IκBα, and p65, a subunit of NF‐κB, was detected both in senescent primary rat AEC2 and in A549. PTEN was significantly reduced along with bleomycin increasing. The phosphorylation levels of IKKβ at serine residues 177 and 181 and IκBα at serine residue 32 were remarkably elevated (Figure 4a–d). Immunofluorescence assays were conducted to test the location and activation of NF‐κB after treatment with bleomycin, increased nuclear localization and activation were observed along with bleomycin accumulation in both rat primary AEC2 and A549 (Figure 4e,f). SP‐C, a specific AEC2 marker, was used to confirm the phenotype of AEC2. We found that SP‐C was not reduced with time within 5 days, suggesting that the cells maintained AEC2 characteristics without switching to AEC1 (Figure 4g). These data suggested that the loss of PTEN activated NF‐κB to promote AEC senescence and the occurrence of SASP, thus participating in the pathogenesis of IPF. To examine this issue, we further conducted a set of in vitro experiments to elucidate the possible mechanisms of cellular senescence in IPF.

### 2.5 NF‐κB silence rescues AECs from senescence and fibroblasts from collagen deposition

**Figure 5:**
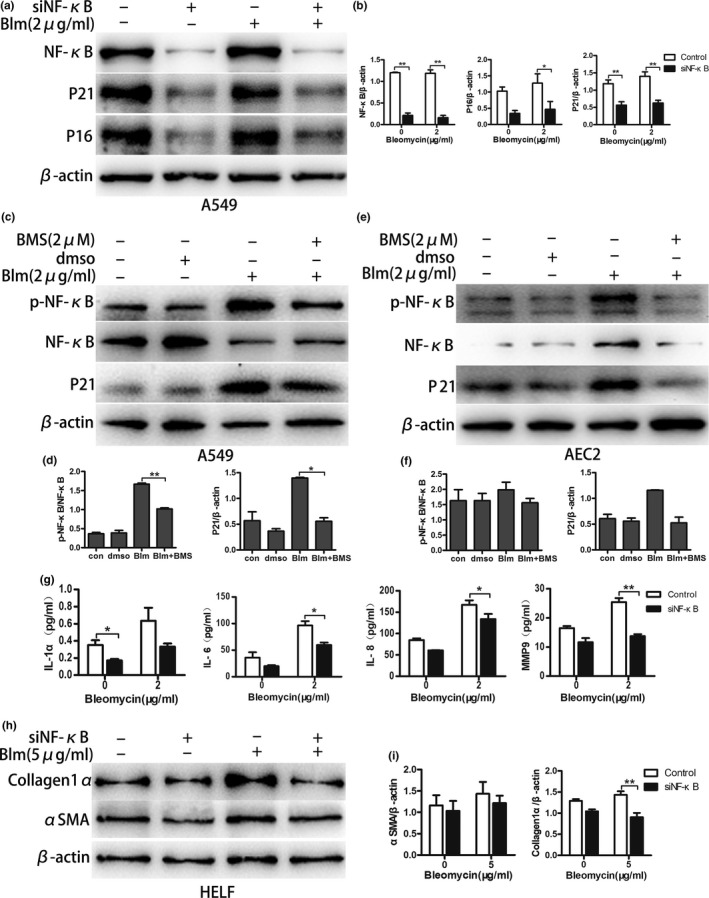
Genetic knockdown and a specific inhibitor of NF‐κB attenuate bleomycin‐induced AEC senescence, SASP and collagen deposition in HELF**.** (a, b) Lentivirus harboring siRNA of NF‐κB was transfected to knock down its expression in A549, followed by bleomycin (2 µg/ml) stimulation for 5 days. The alterations of P16^ink4a^ and P21^WAF1^ expression were investigated. (c,d,e, and f) NF‐κB‐specific inhibitor BMS‐345541 (2 µM) dissolved in DMSO was added together with bleomycin (2 µg/ml) in the culture medium and treated A549 and AEC2 for 5 days; NF‐κB activation and P21^WAF1^ expression was detected by western blot. (g) After NF‐κB knockdown in A549, they were stimulated by bleomycin (2 µg/ml) for 5 days, and then the supernatants were detected by ELISA to examine alterations of IL‐1α, IL‐6, IL‐8, and MMP9. (h, i) Bleomycin (5 µg/ml) was added in NF‐κB knocked‐down A549 culture medium for 3 days, then the medium was replaced by fresh bleomycin‐free medium to culture another 3 days. Finally, the supernatants were collected to foster HELF for 3 days and the expression of αSMA and collagen1α was measured. Data are shown as the mean ± *SEM*, *n* ≥ 3 per group. *p < 0.05, **p < 0.005. One‐way ANOVA analysis followed by Dunnett's Multiple Comparison Test or two‐way ANOVA analysis followed by Bonferroni posttests

The activation of NF‐κB is thought to accelerate cellular senescence and control SASP. NF‐κB inhibition delays DNA damage‐induced senescence and aging in mice (Salminen et al., 2012; Tilstra et al., 2012). Similarly, we observed the activation of NF‐κB in senescent AECs. We then verified whether activated NF‐κB had a regulatory effect on AEC senescence and SASP and on triggering the deposition of collagen in fibroblasts. To elucidate this issue, the expression of NF‐κB was knocked down, and a specific inhibitor of the NF‐κB pathway was used. As shown in Figure 5, senescent markers P16^ink4a^ and P21^WAF1^ and SASP‐associated markers were reduced after the genetic manipulation of NF‐κB (Figure 5a,b,g). In addition, after the inhibition of NF‐κB activation using BMS‐345541 (an IKKα/β‐specific inhibitor), the expression of P21^WAF1^ was decreased in both A549 and AEC2 (Figure 5c–f). Then, to investigate whether the deposition of collagen in fibroblasts could be triggered by SASP through NF‐κB activation, the senescent supernatants of A549 cells, after blocking NF‐κB activation, were collected to stimulate HELF for 3 days, and then, collagen 1α and α‐SMA were measured by western blot. Interestingly, we observed that collagen 1α expression was decreased in total HELF protein lysates stimulated by senescent supernatants in which the NF‐κB activity was blocked in A549 (Figure 5h,i). Taken together, after the genetic knockdown of NF‐κB, senescent markers of AECs were lower and the expression of fibrotic markers was lower in HELF than in the control groups. These results provide evidence that the activation of NF‐κB participates in AEC senescence and the deposition of collagen in HELF in the pathogenetic process of IPF.

### 2.6 Deletion of PTEN accelerates AEC senescence and augments collagen deposition in fibroblasts via NF‐κB activation

**Figure 6:**
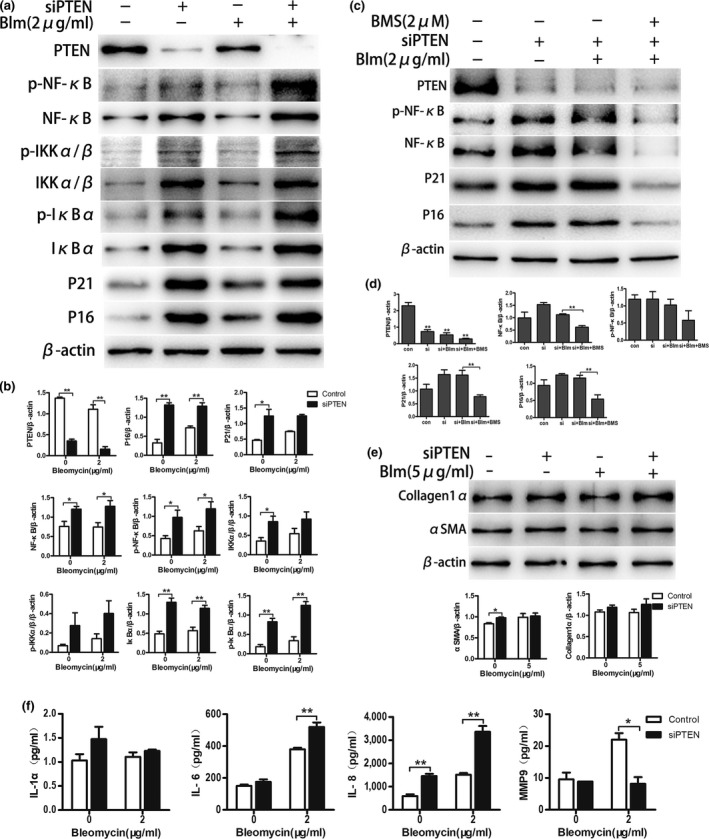
Loss of PTEN drives AECs senescence and promotes collagen accumulation in HELF via the activation of NF‐κB. (a, b) Lentivirus vector was used to knock down expression of PTEN in A549, followed by bleomycin (2 µg/ml) stimulation for 5 days. Western blot was performed to confirm the alteration of P16^ink4a^, P21^WAF1,^ and targets of PTEN/NF‐κB pathway in total protein extracts. (c, d) After PTEN was knocked down, an inhibitor of the NF‐κB pathway (BMS‐345541) was added together with bleomycin (2 µg/ml) to culture A549 for 5 days, NF‐κB activation and P16^ink4a^, P21^WAF1^ expression were detected by western blot. (e) Bleomycin (5 µg/ml) was added in culture medium of PTEN knockdown A549 for 3 days, the supernatants were collected to culture HELF for 3 days and western blotting was performed to detect fibrotic markers (collagen 1α and αSMA). (f) After PTEN knockdown in A549, cells were stimulated by bleomycin (2 µg/ml) for 5 days, the supernatants were collected, and SASP markers were measured by ELISA. Data are shown as the mean ± *SEM*, *n* ≥ 3 per group. *p < 0.05, **p < 0.005. One‐way ANOVA analysis followed by Dunnett's Multiple Comparison Test or two‐way ANOVA analysis followed by Bonferroni posttests

Although our initial experiments showed reduced PTEN expression and elevated NF‐κB activation in IPF lung tissues and in in vitro experiments, it remained uncertain whether decreased PTEN and increased NF‐κB activation were responsible for the induction of AEC senescence. To determine whether the loss of PTEN accelerates AEC senescence through the activation of NF‐κB, PTEN gene expression knockdown was performed in A549. Cells were cultured in medium with bleomycin for 5 days, and total protein lysates were collected to perform western blotting. As shown in Figure 6, we found that the NF‐κB pathway was activated after PTEN knockdown, and the expression levels of aging‐related markers P21^ink4a^ and P16^WAF1^ and SASP markers were substantially higher in the knockdown cells than in the control cells (Figure 6a,b,f). Then, we explored whether the accelerated senescence of AECs triggered by loss of PTEN could be reversed by NF‐κB inhibition. BMS‐345541 was applied to inhibit the activation of NF‐κB in PTEN knockdown A549. As expected, P16^ink4a^ and P21^WAF1^ were significantly lower after treatment with BMS‐345541 (Figure 6c,d). To further delineate the underlying relationship between the loss of PTEN and lung fibrosis, the senescent supernatants from PTEN knockdown A549 cells were collected to culture HELF for 3 days. As shown in Figure 6e, the senescent supernatants from PTEN knockdown A549 increased the collagen 1α and α‐SMA expression of HELF, but the results partly lacked statistical significance. Together, these results support our hypothesis that the loss of PTEN accelerates the senescence of AECs via the activation of the NF‐κB pathway and induces collagen deposition in fibroblasts through the SASP of senescent AECs.

### 2.7 Cellular senescence and the activated PTEN/NF‐κB pathway in the bleomycin‐induced mouse pulmonary fibrosis model

**Figure 7:**
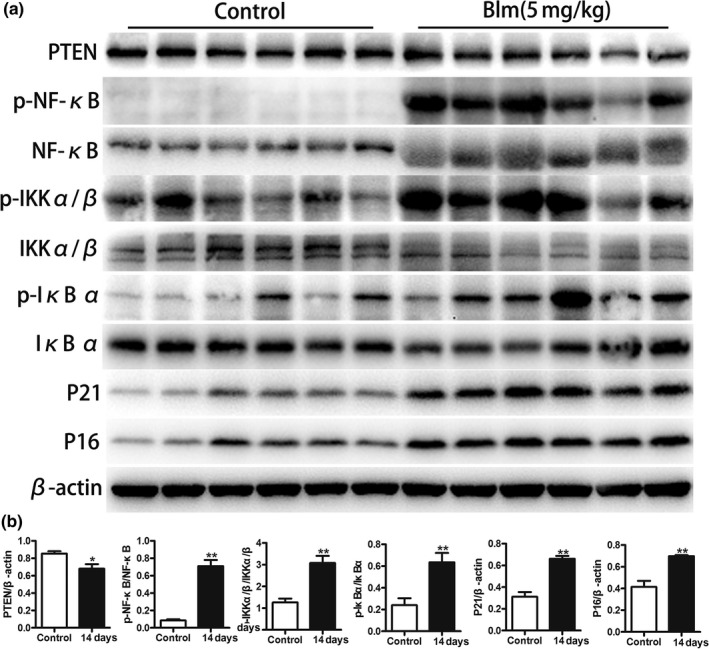
Increased senescence markers and the activation of the PTEN/NF‐κB pathway in the bleomycin‐induced mouse pulmonary fibrosis model. (a, b) C57BL/6 mice were randomly divided into 0.9% saline water group (*n* = 6) and the bleomycin group (*n* = 6). Mice were intratracheally injected with 50 µl 0.9% saline and 50 µl 5 mg/kg bleomycin, respectively. They were both sacrificed, and lungs were collected at 14 days after manipulation. Total proteins were extracted from mouse lung tissues, and western blotting was performed to detect senescent markers (P21^WAF1^ and P16^ink4a^) as well as the expression of the PTEN/NF‐κB pathway. Data are shown as the mean ± *SEM*, *p < 0.05, **p < 0.005. Unpaired, two‐tailed Student's *t* test

A bleomycin‐induced mouse pulmonary fibrosis model was also developed. As shown in Figure 7, the expression levels of senescence‐related markers P21^WAF1^ and P16 ^ink4a^ were significantly higher in the experimental group than in the control group (Figure 7a,b), indicating that cellular senescence also occurred in mouse lung tissue after treatment with bleomycin. Simultaneously, the decreased expression of PTEN and activated NF‐κB pathway were also detected in the bleomycin group (Figure 7a,b).

## 3 DISCUSSION

We confirmed that AECs developed accelerated cellular senescence in IPF, supporting the notion that IPF is an aging‐related disease. These findings agree with those of recent studies (Chilosi et al., 2013; Miyoshi et al., 2013; Schafer et al., 2017; Sueblinvong et al., 2012). In the present study, we made a novel finding that the PTEN/NF‐κB pathway participated in the pathogenesis of pulmonary fibrosis, primarily by regulating the senescence of AECs. Another striking finding of our study was that the supernatants of senescent AECs directly triggered collagen deposition in fibroblasts, partially clarifying the interaction between AEC senescence and fibroblasts.

Low levels of PTEN expression in fibroblasts and epithelial cells from IPF lungs have been detected. Mice with a constitutive deficiency of PTEN develop spontaneous fibrosis via intrinsic defects in fibroblasts and the negative regulation of their proliferative responses (Parapuram et al., 2015). Furthermore, the conditioned targeted deletion of PTEN in the lung epithelium exacerbates injury and fibrosis after bleomycin treatment via an increase in active TGF‐β and the loss of epithelial integrity (Miyoshi et al., 2013). In fibroblasts isolated from IPF patients, decreased caveolin‐1, an integral membrane protein, correlates with low membrane PTEN levels, and decreased caveolin‐1 may down‐regulate PTEN expression to participate in pathogenesis of IPF (Xia et al., 2010). Identical to the previous findings, our study observed that PTEN also expressed in AEC2 in human lung tissues and its expression was much lower in IPF lung tissues than in healthy control (Figure 2c,d). These findings support that the role of decreased PTEN may play an important role in regulation of cell growth status during AEC injury.

PTEN is closely related to cellular senescence in various human diseases (Ahmad et al., 2011; Moon et al., 2013). For example, during the progression of replicated skin aging, PTEN is down‐regulated (Noh et al., 2016). In contrast, the loss of PTEN in non‐tumor‐forming β‐cells leads to the down‐regulation of P16^ink4a^ expression, and this effect partially blocks the aging‐related loss of cell proliferation capacity (Zeng et al., 2013). These studies indicate that PTEN regulates cellular senescence in a cell type‐specific way. In an animal model, increased PTEN gene expression prolongs the mouse lifespan, and its down‐regulation leads to accelerated cell senescence; these results reveal that PTEN has a negative regulatory role in aging (Ortega‐Molina & Serrano, 2013; Ortega‐Molina et al., 2012). In our study, we also observed that the loss of PTEN accelerated the senescence of AECs in pulmonary fibrosis.

PTEN occurs primarily through the negatively regulation of PI3K/Akt activation to exert its functions, and NF‐κB is an important target of the PI3K/Akt pathway to regulate the secretion of many cytokines (Cheng et al., 2014). PTEN physically interacts with IKK, as the PTEN‐IKK complex, preventing its phosphorylation and thus inhibiting the activation of NF‐κB. PTEN‐overexpressed cells are unable to phosphorylate IKK (Zaidi & Manna, 2016). In macrophage isolated from bovine lung, PTEN negatively regulates NF‐κB activation and thus affects the production of cytokines under the stimulation of lipopolysaccharide (LPS), suggesting as an upstream regulator, PTEN can negatively regulate NF‐κB‐related inflammatory process, and this effect of PTEN may be controlled by miR‐26b (Zhang, Huang, Guo, & Gou, 2015). In our study, decreased PTEN and activated NF‐κB pathway were also observed in lung tissues from IPF patients and lung fibrotic mouse, and senescent AECs induced by bleomycin stimulation. Gene silence of PTEN could accelerate the senescence of AEC via NF‐κB activation. We also found that senescence of the AECs controlled by PTEN‐regulated NF‐κB activity may further affect the adjacent environments which was proved by increased collagen deposition in fibroblast cultured with the supernatant of senescent AEC. In together, PTEN can suppress NF‐κB activity and participate in inflammatory processes within AECs.

NF‐κB is an important target of the PTEN/PI3K/Akt pathway, regulating the secretion of numerous cytokines (Cheng et al., 2014). Moreover, the activation of NF‐κB participates in the aging process to promote cellular senescence in various kinds of cells (Donato et al., 2007; Hasegawa et al., 2012; Rovillain et al., 2011). Its signaling transduction triggers cytokine release in senescent cells, known as SASP, thereby contributing to the adjacent cellular microenvironment (Salminen et al., 2012). Through the secretion of the SASP, including the release of a broad repertoire of cytokines and chemokines, changes in the expression of matrix remodeling proteases and growth factors, senescent cells regulate the microenvironment in a paracrine manner, by promoting cell proliferation and tissue degradation, modifying inflammation, tissue repair, fibrosis, and carcinogenesis. For example, SASP cytokines, and increased levels of MMPs, such as MMP‐2 and MMP‐9, participate in the development of bleomycin‐induced lung injury and fibrosis, and the NF‐κB and p38 MAPK pathways account for SASP regulation (Alimbetov et al., 2016; Aoshiba et al., 2013). In accordance with previous studies, our findings support the notion that NF‐κB‐controlled SASP release from senescent AECs confers fibrotic phenotypes in fibroblasts, suggesting that senescence in AECs can work as an initiating factor boosting the development and progression of IPF. To the best of our knowledge, this is the first clear indication that the senescence of AECs mediated by the PTEN/NF‐κB pathway is involved in IPF pathogenesis via the manipulation of collagen deposition in fibroblasts. These findings might shed light on the possibility of the intervention or disruption of IPF progression at very early stages through interference with AEC senescence.

Most recently, it suggested that elimination of senescent cells using senolytic drugs could relieve or reverse the development of aging‐related chronic disease, such as cardiac dysfunction, diabetes mellitus, vertebral disk degeneration, pulmonary fibrosis, and radiation‐induced tissue damage (Kirkland et al., 2017). In fibrotic AEC2 cells and a three‐dimensional lung tissue cultures model, eliminating senescent epithelial cells using senolytic drugs could reduce the release of SASP factors and the production of extracellular matrix, suggesting that senescence of AEC2 cells could confer the environment a profibrotic phenotype. And anti‐senescence treatment might be beneficial to blocking the process of pulmonary fibrosis (Lehmann et al., 2017). In accordance with Lehmann's study, senescence of epithelial cells was also found in human lung fibrotic tissue in the current study. It also proved that the senescence of AECs could change their secretory phenotypes and thus might exhibit as a profibrotic initiator to the adjacent cells and tissue. And PTEN/NF‐κB pathway may be a novel candidate for senolytic drugs which may advance treatment of IPF.

In conclusion, AEC senescence controlled by the PTEN/NF‐κB pathway is a characteristic of lung fibrosis, and the release of SASP from senescent AECs affects the niches around them, thus driving the process of fibrosis by promoting collagen deposition in fibroblasts. And interactions between epithelial and fibroblast may be an important factor to induce the genesis and development of IPF.

## 4 MATERIALS AND METHODS

### 4.1 Cell lines

A549 and HELF were purchased from American Type Culture Collection (Rockville, MD, USA). They were all cultured in DMEM (Gibco, Australia), supplemented with 10% FBS (Gibco) and 1X penicillin–streptomycin solution (Thermo Fisher Scientific, USA), in a 37°C humidified 5% CO_2_ incubator. Human Pulmonary Fibroblasts‐adult (HPF‐a, Cat. #3,310) was purchased from ScienCell Research Laboratories (San Diego, CA, USA),and it was isolated from adult human lung tissue. And it was characterized by immunofluorescence staining to Vimentin, and its positive rate was over 90%, the purity of the cells was over 90%. And HPF‐a was cultured in specific fibroblast medium (Cat. #2,301) under the manufacturer's instructions.

### 4.2 Reagents

Bleomycin, purchased from Nippon Kayaku Co Ltd. (Japan), was dissolved in 0.9% saline and stored at −20°C. BMS‐345541, an inhibitor of both IKK‐1 and IKK‐2, was purchased from Selleck Chemicals (USA) and dissolved in DMSO (Sigma, USA), stored at 4°C.

### 4.3 Primary type 2 alveolar epithelial cell (AEC2) isolation and culture

After anesthesia, the lungs of *SD* rats were excised, washed in PBS, and cut into pieces. Then, trypsin was added to digest the tissues for 30 min at 37°C, and the reaction was terminated with DMEM containing 10% FBS. The tissues were struck repeatedly to separate AECs. After washing twice in PBS, the cell suspension was transferred into culture flasks coated with rat IgG‐supplemented complete medium. After 2 hr, the detached cells were removed by centrifugation, and the supernatants were discarded. Pellets were suspended in complete medium and cultured in a 37°C humidified incubator containing 5% CO_2_. Finally, IF staining for SP‐C (Santa Cruz, CA, USA) was conducted to identify ACE2 cells.

### 4.4 Human patient material

All human lung tissues of IPF (*n* = 12) were obtained from the Department of Lung Transplantation, Wuxi People's Hospital, Wuxi, China. Normal peripheral tissues (*n* = 12) from tumor patients supplied by the Thoracic Surgery Department of Nanjing Drum Tower Hospital of the Affiliated Hospital of Nanjing University Medical School used as controls. All diagnoses of IPF were made in accordance with the ATS/ERS criteria for IPF 2011. Twelve patients who provided normal lung tissues, the average age was 63.16 ± 4.62 (mean ± *SEM*) and including 1 female patient and 11 male patients. And 12 IPF patients, their average age was 61.83 ± 5.49 (mean ± *SEM*) and including one female patient and 11 male patients. Informed consent was obtained from patients, and the study is officially approved by the Ethics Committee of Medical School of Nanjing University.

### 4.5 Mouse pulmonary fibrosis models

Six‐ to eight‐week‐old male SPF C57BL/6 mice (Shanghai Laboratory Animal Center, Chinese Academy of Sciences, Shanghai, China) were randomly divided into the vehicle group (*n* = 6) and treated group (*n* = 6). Mice in the vehicle group were intratracheally injected with 50 µl 0.9% saline, and the treated group was injected intratracheally with 50 µl 5 mg/kg bleomycin. On day 14 after bleomycin or saline treatment, mice were sacrificed, and lungs were collected for subsequent experiments. All procedures involving animals were approved by the Ethics Committee for Animal Research of Medical School of Nanjing University.

### 4.6 Cellular aging model

A549 and AEC2 were stimulated with specific concentrations of bleomycin for 5 days to build the cellular senescence model. Then, several aging‐related markers were tested, including p16, p21, senescence‐associated β‐galactosidase (SA‐β‐Gal), and SASP, to confirm the senescent state of the cells.

### 4.7 Lentivirus transfection

To build stable genetic silencing of PTEN and NF‐κB, lentivirus was used as a vector to carry the interference sequence. The lentivirus vectors loaded with the targeting gene and nontargeting control were constructed by GenChem, Shanghai, China. Then, lentivirus was added to 1 ml complete medium per well of a 6‐well slide supplemented with 5 µg/ml polybrene. After 8–12 hr, the medium was replaced with fresh complete medium without lentivirus and polybrene to culture for another 72 hr. Green fluorescence was observed for rough judgments of transfection efficiency, and total cellular protein was extracted to further confirm the transfection efficiency by western blotting.

### 4.8 Senescence‐associated β‐galactosidase staining

SA‐β‐Gal staining was performed according to the manufacturer's protocol. The Senescence β‐Galactosidase Staining Kit was purchased from Beyotime Biotechnology (Shanghai, China). Cell samples on 6‐well chamber slides and frozen lung tissue sections were fixed with 4% formaldehyde for 10 min at room temperature. The slides were rinsed three times with PBS for 5 min and then incubated with freshly prepared SA‐β‐Gal staining solution overnight in a 37°C humidified chamber. The next day, the slide and tissue sections were washed twice in PBS for 10 min at room temperature. To better visualize alveolar structure, the tissue sections were further counterstained with eosin. Then, they were observed, and pictures were captured using a microscope equipped with a digital camera (Eclipse e800, Nikon). For each slide, at least three fields were captured to calculate the SA‐β‐gal intensity.

### 4.9 Western blot

Whole protein from cell lysates and lung tissues was extracted according to manufacturer's instructions (Keygene, China). Protein samples were separated on 10% SDS‐PAGE gels, and then, the proteins were transferred to PVDF membranes (Merck Millipore, Germany), blocked with 5% nonfat milk in TBST and incubated with primary antibody at room temperature for 4 hr or overnight. Then, membranes were incubated with HRP‐conjugated secondary antibody for one hour, and protein expression was detected using ECL (Merck Millipore, Germany). Primary antibodies against P16, P21, αSMA, and Collagen1α were purchased from Abcam (United Kingdom). Antibodies against PTEN, IKK, phospho‐IKK, IκB, phospho‐IκB, NF‐κB, and phospho‐NF‐κB were purchased from Cell Signaling Technology (USA).

### 4.10 Immunohistochemical staining

The tissues were fixed in formalin and embedded in paraffin. Immunohistochemical staining for target proteins and hematoxylin–eosin staining (HE) were performed. Four‐micron‐thick sections were deparaffinized in xylene and rehydrated in graded alcohol. Endogenous peroxidase was quenched with 3% aqueous hydrogen peroxide for 15 min. Then, antigen retrieval was performed in a pressure cooker. After primary antibody incubation overnight at 4°C, horseradish peroxidase‐conjugated secondary antibody was added for 20 min, and 3,3‐diaminobenzidine tetrahydrochloride (DAB) was added for 10 min at room temperature. Finally, sections were dehydrated and mounted.

### 4.11 Immunofluorescence assay

Four percent paraformaldehyde‐fixed, paraffin‐embedded blocks of lung tissues were cut into 4‐μm sections. After deparaffinating, rehydration and retrieval, tissues were incubated with primary antibodies overnight at 4°C. Cells in 24‐hole chambers were fixed in 4% paraformaldehyde for 10 min and blocked with 0.2% Triton X‐100 dissolved in 1% BSA for 30 min. Then, cells were incubated with primary antibodies overnight at 4°C. After rinsing several times with PBS, cells were incubated with Alexa‐488‐ and Alexa‐568‐conjugated secondary antibodies (Invitrogen, United Kingdom) for 2 hr. Finally, DAPI was added to the wells for 10 min. Images were obtained with a ZEISS Imager.A1 fluorescence microscope (Gottingen, Germany). Immunofluorescence was performed using the following primary antibodies: Rabbit monoclonal antibodies, anti‐P21 and anti‐PTEN, were purchased from Abcam (United Kingdom). Mouse monoclonal antibody, anti‐SP‐C, was purchased from Santa Cruz Biotechnology (CA, USA).

### 4.12 ELISA

The levels of IL‐1, IL‐6, IL‐8, and MMP9 in supernatants were measured using commercially available ELISA kits (Raybiotech, USA), and experiments were conducted according to the manufacturers’ instructions.

### 4.13 Statistical analysis

Statistical analysis was performed using Prism 7 (graphpad). Differences between groups were calculated by two‐tailed Student's *t* test or by one‐ or two‐way ANOVA analysis followed by post hoc tests. These data are presented as the mean ± *SEM*. The results are considered statistically significant if *p* < 0.05.

## CONFLICT OF INTEREST

All authors declare no potential conflict of interests.

## AUTHORS' CONTRIBUTIONS

Yaqiong Tian, Hui Li, Jinghong Dai, Yingwei Zhang, and Hourong Cai took charge of paper writing, designing, and performing the experiments. Ting Qiu was primarily responsible for data analysis and creating figures. Jingyu Chen was primarily responsible for making experiment plans and supplying some of the human specimens.

## References

[acel12858-bib-0001] Ahmad, I. , Patel, R. , Singh, L. B. , Nixon, C. , Seywright, M. , Barnetson, R. J. , … Leung, H. Y. (2011). HER2 overcomes PTEN (loss)‐induced senescence to cause aggressive prostate cancer. Proceedings of the National Academy of Sciences of the United States of America, 108(39), 16392–16397. 10.1073/pnas.1101263108. 21930937 PMC3182686

[acel12858-bib-0002] Alimbetov, D. , Davis, T. , Brook, A. J. , Cox, L. S. , Faragher, R. G. , Nurgozhin, T. , … Kipling, D. (2016). Suppression of the senescence‐associated secretory phenotype (SASP) in human fibroblasts using small molecule inhibitors of p38 MAP kinase and MK2. Biogerontology, 17(2), 305–315. 10.1007/s10522-015-9610-z. 26400758 PMC4819486

[acel12858-bib-0003] Aoshiba, K. , Tsuji, T. , Kameyama, S. , Itoh, M. , Semba, S. , Yamaguchi, K. , & Nakamura, H. (2013). Senescence‐associated secretory phenotype in a mouse model of bleomycin‐induced lung injury. Experimental and Toxicologic Pathology, 65(7–8), 1053–1062. 10.1016/j.etp.2013.04.001. 23688655

[acel12858-bib-0004] Aoshiba, K. , Tsuji, T. , & Nagai, A. (2003). Bleomycin induces cellular senescence in alveolar epithelial cells. European Respiratory Journal, 22(3), 436–443. 10.1183/09031936.03.00011903 14516132

[acel12858-bib-0005] Buendía‐Roldán, I. , Mejía, M. , Navarro, C. , & Selman, M. (2017). Idiopathic pulmonary fibrosis: Clinical behavior and aging associated comorbidities. Respiratory Medicine, 129, 46–52. 10.1016/j.rmed.2017.06.001 28732835

[acel12858-bib-0006] Cheng, S. E. , Lee, I. T. , Lin, C. C. , Hsiao, L. D. , & Yang, C. M. (2014). Thrombin induces ICAM‐1 expression in human lung epithelial cells via c‐Src/PDGFR/PI3K/Akt‐dependent NF‐kappaB/p300 activation. Clinical Science (London), 127(3), 171–183. 10.1042/cs20130676. 24506791

[acel12858-bib-0007] Chilosi, M. , Carloni, A. , Rossi, A. , & Poletti, V. (2013). Premature lung aging and cellular senescence in the pathogenesis of idiopathic pulmonary fibrosis and COPD/emphysema. Translational Research, 162(3), 156–173. 10.1016/j.trsl.2013.06.004. 23831269

[acel12858-bib-0008] Coppe, J. P. , Desprez, P. Y. , Krtolica, A. , & Campisi, J. (2010). The senescence‐associated secretory phenotype: The dark side of tumor suppression. Annual Review of Pathology: Mechanisms of Disease, 5, 99–118. 10.1146/annurev-pathol-121808-102144. PMC4166495 20078217

[acel12858-bib-0009] Donato, A. J. , Eskurza, I. , Silver, A. E. , Levy, A. S. , Pierce, G. L. , Gates, P. E. , & Seals, D. R. (2007). Direct evidence of endothelial oxidative stress with aging in humans: Relation to impaired endothelium‐dependent dilation and upregulation of nuclear factor‐kappaB. Circulation Research, 100(11), 1659–1666. 10.1161/01.RES.0000269183.13937.e8. 17478731

[acel12858-bib-0010] Hasegawa, Y. , Saito, T. , Ogihara, T. , Ishigaki, Y. , Yamada, T. , Imai, J. , … Katagiri, H. (2012). Blockade of the nuclear factor‐kappaB pathway in the endothelium prevents insulin resistance and prolongs life spans. Circulation, 125(9), 1122–1133. 10.1161/circulationaha.111.054346. 22302838

[acel12858-bib-0011] Kasper, M. , & Barth, K. (2009). Bleomycin and its role in inducing apoptosis and senescence in lung cells ‐ modulating effects of caveolin‐1. Current Cancer Drug Targets, 9(3), 341–353. 19442053 10.2174/156800909788166501

[acel12858-bib-0012] King, T. E. Jr , Pardo, A. , & Selman, M. (2011). Idiopathic pulmonary fibrosis. Lancet, 378(9807), 1949–1961. 10.1016/s0140-6736(11)60052-4. 21719092

[acel12858-bib-0013] Kirkland, J. L. , Tchkonia, T. , Zhu, Y. , Niedernhofer, L. J. , & Robbins, P. D. (2017). The clinical potential of senolytic drugs. Journal of the American Geriatrics Society, 65(10), 2297–2301. 10.1111/jgs.14969. 28869295 PMC5641223

[acel12858-bib-0014] Kuilman, T. , & Peeper, D. S. (2009). Senescence‐messaging secretome: SMS‐ing cellular stress. Nature Reviews Cancer, 9(2), 81–94. 10.1038/nrc2560. 19132009

[acel12858-bib-0015] Lehmann, M. , Korfei, M. , Mutze, K. , Klee, S. , Skronska‐Wasek, W. , Alsafadi, H. N. , … Konigshoff, M. (2017). Senolytic drugs target alveolar epithelial cell function and attenuate experimental lung fibrosis ex vivo. European Respiratory Journal, 50(2), 10.1183/13993003.02367-2016. PMC5593348 28775044

[acel12858-bib-0016] Ley, B. , & Collard, H. R. (2013). Epidemiology of idiopathic pulmonary fibrosis. Clinical Epidemiology, 5, 483–492. 10.2147/clep.s54815. 24348069 PMC3848422

[acel12858-bib-0017] Minagawa, S. , Araya, J. , Numata, T. , Nojiri, S. , Hara, H. , Yumino, Y. , Kuwano, K. (2011). Accelerated epithelial cell senescence in IPF and the inhibitory role of SIRT6 in TGF‐beta‐induced senescence of human bronchial epithelial cells. American Journal of Physiology. Lung Cellular and Molecular Physiology, 300(3), L391–L401. 10.1152/ajplung.00097.2010. 21224216 PMC3284316

[acel12858-bib-0018] Miyoshi, K. , Yanagi, S. , Kawahara, K. , Nishio, M. , Tsubouchi, H. , Imazu, Y. , … Nakazato, M. (2013). Epithelial Pten controls acute lung injury and fibrosis by regulating alveolar epithelial cell integrity. American Journal of Respiratory and Critical Care Medicine, 187(3), 262–275. 10.1164/rccm.201205-0851OC. 23239155

[acel12858-bib-0019] Moon, S. H. , Kim, D. K. , Cha, Y. , Jeon, I. , Song, J. , & Park, K. S. (2013). PI3K/Akt and Stat3 signaling regulated by PTEN control of the cancer stem cell population, proliferation and senescence in a glioblastoma cell line. International Journal of Oncology, 42(3), 921–928. 10.3892/ijo.2013.1765. 23314408

[acel12858-bib-0020] Noh, E. M. , Park, J. , Song, H. R. , Kim, J. M. , Lee, M. , Song, H. K. , … Lee, Y. R. (2016). Skin aging‐dependent activation of the PI3K signaling pathway via downregulation of PTEN increases intracellular ROS in human dermal fibroblasts. Oxidative Medicine and Cellular Longevity, 2016, 6354261 10.1155/2016/6354261. 28003865 PMC5149682

[acel12858-bib-0021] Ortega‐Molina, A. , Efeyan, A. , Lopez‐Guadamillas, E. , Munoz‐Martin, M. , Gomez‐Lopez, G. , Canamero, M. , … Serrano, M. (2012). Pten positively regulates brown adipose function, energy expenditure, and longevity. Cell Metabolism, 15(3), 382–394. 10.1016/j.cmet.2012.02.001. 22405073

[acel12858-bib-0022] Ortega‐Molina, A. , & Serrano, M. (2013). PTEN in cancer, metabolism, and aging. Trends in Endocrinology and Metabolism, 24(4), 184–189. 10.1016/j.tem.2012.11.002. 23245767 PMC3836169

[acel12858-bib-0023] Parapuram, S. K. , Thompson, K. , Tsang, M. , Hutchenreuther, J. , Bekking, C. , Liu, S. , & Leask, A. (2015). Loss of PTEN expression by mouse fibroblasts results in lung fibrosis through a CCN2‐dependent mechanism. Matrix Biology, 43, 35–41. 10.1016/j.matbio.2015.01.017. 25644104

[acel12858-bib-0024] Rovillain, E. , Mansfield, L. , Caetano, C. , Alvarez‐Fernandez, M. , Caballero, O. L. , Medema, R. H. , … Jat, P. S. (2011). Activation of nuclear factor‐kappa B signalling promotes cellular senescence. Oncogene, 30(20), 2356–2366. 10.1038/onc.2010.611. 21242976 PMC3080811

[acel12858-bib-0025] Salminen, A. , Kauppinen, A. , & Kaarniranta, K. (2012). Emerging role of NF‐kappaB signaling in the induction of senescence‐associated secretory phenotype (SASP). Cellular Signalling, 24(4), 835–845. 10.1016/j.cellsig.2011.12.006. 22182507

[acel12858-bib-0026] Schafer, M. J. , White, T. A. , Iijima, K. , Haak, A. J. , Ligresti, G. , Atkinson, E. J. , … LeBrasseur, N. K. (2017). Cellular senescence mediates fibrotic pulmonary disease. Nature Communications, 8, 14532 10.1038/ncomms14532. PMC5331226 28230051

[acel12858-bib-0027] Solt, L. A. , & May, M. J. (2008). The IkappaB kinase complex: Master regulator of NF‐kappaB signaling. Immunologic Research, 42(1–3), 3–18. 10.1007/s12026-008-8025-1. 18626576 PMC2965074

[acel12858-bib-0028] Sueblinvong, V. , Neujahr, D. C. , Mills, S. T. , Roser‐Page, S. , Ritzenthaler, J. D. , Guidot, D. , … Roman, J. (2012). Predisposition for disrepair in the aged lung. American Journal of the Medical Sciences, 344(1), 41–51. 10.1097/MAJ.0b013e318234c132. 22173045 PMC3395069

[acel12858-bib-0029] Tilstra, J. S. , Robinson, A. R. , Wang, J. , Gregg, S. Q. , Clauson, C. L. , Reay, D. P. , … Robbins, P. D. (2012). NF‐kappaB inhibition delays DNA damage‐induced senescence and aging in mice. Journal of Clinical Investigation, 122(7), 2601–2612. 10.1172/jci45785. 22706308 PMC3386805

[acel12858-bib-0030] White, E. S. , Atrasz, R. G. , Hu, B. , Phan, S. H. , Stambolic, V. , Mak, T. W. , … Toews, G. B. (2006). Negative regulation of myofibroblast differentiation by PTEN (phosphatase and tensin homolog deleted on chromosome 10). American Journal of Respiratory and Critical Care Medicine, 173(1), 112–121. 10.1164/rccm.200507-1058OC. 16179636 PMC1434700

[acel12858-bib-0031] Xia, H. , Khalil, W. , Kahm, J. , Jessurun, J. , Kleidon, J. , & Henke, C. A. (2010). Pathologic caveolin‐1 regulation of PTEN in idiopathic pulmonary fibrosis. American Journal of Pathology, 176(6), 2626–2637. 10.2353/ajpath.2010.091117. 20395445 PMC2877826

[acel12858-bib-0032] Yanagi, S. , Kishimoto, H. , Kawahara, K. , Sasaki, T. , Sasaki, M. , Nishio, M. , … Suzuki, A. (2007). Pten controls lung morphogenesis, bronchoalveolar stem cells, and onset of lung adenocarcinomas in mice. Journal of Clinical Investigation, 117(10), 2929–2940. 10.1172/jci31854. 17909629 PMC1994617

[acel12858-bib-0033] Yanagi, S. , Tsubouchi, H. , Miura, A. , Matsumoto, N. , & Nakazato, M. (2015). Breakdown of epithelial barrier integrity and overdrive activation of alveolar epithelial cells in the pathogenesis of acute respiratory distress syndrome and lung fibrosis. BioMed Research International, 2015, 573210 10.1155/2015/573210. 26523279 PMC4615219

[acel12858-bib-0034] Zaidi, A. H. , & Manna, S. K. (2016). Profilin‐PTEN interaction suppresses NF‐kappaB activation via inhibition of IKK phosphorylation. The Biochemical Journal, 473(7), 859–872. 10.1042/bj20150624. 26787927

[acel12858-bib-0035] Zeng, N. , Yang, K. T. , Bayan, J. A. , He, L. , Aggarwal, R. , Stiles, J. W. , … Stiles, B. L. (2013). PTEN controls beta‐cell regeneration in aged mice by regulating cell cycle inhibitor p16ink4a. Aging Cell, 12(6), 1000–1011. 10.1111/acel.12132. 23826727 PMC3838454

[acel12858-bib-0036] Zhang, L. , Huang, C. , Guo, Y. , & Gou, X. (2015). MicroRNA‐26b modulates the NF‐kappaB pathway in alveolar macrophages by regulating PTEN. Journal of Immunology, 195(11), 5404–5414. 10.4049/jimmunol.1402933. PMC4655123 26503952

